# Advancing the Development of the China‐SCO Cooperation Center for Metabolic Diseases to Foster a Global Community of Health for All

**DOI:** 10.1111/1753-0407.70244

**Published:** 2026-06-10

**Authors:** Guang Ning

**Affiliations:** ^1^ Department of Endocrine and Metabolic Diseases Shanghai Institute of Endocrine and Metabolic Diseases, Ruijin Hospital, Shanghai Jiao Tong University School of Medicine Shanghai China; ^2^ Shanghai National Clinical Research Center for Metabolic Diseases, Key Laboratory for Endocrine and Metabolic Diseases of the National Health Commission of the PR China Shanghai National Center for Translational Medicine Shanghai China

At present, metabolic diseases have become a major global public health challenge threatening human health. They not only impose a substantial disease burden on patients and their families, but also present severe challenges to healthcare systems as well as socioeconomic development worldwide.

President Xi Jinping has emphasized the importance of advancing a more just and equitable global governance system and working together toward the building of a global community with a shared future for mankind. At the 24th Meeting of the Council of Heads of Government (Prime Ministers) of the Shanghai Cooperation Organization (SCO) Member States held in Moscow, Premier Li Qiang formally proposed the establishment of the China‐SCO Cooperation Center for Metabolic Diseases. The initiative received positive responses and broad support from all member states. This initiative fully demonstrates China's sense of responsibility as a major country and opens up broad new prospects for health cooperation within the SCO.

Three months ago, the China‐SCO Cooperation Center for Metabolic Diseases was officially inaugurated in Shanghai. Mayor Gong Zheng and SCO Secretary‐General Yermekbayev jointly unveiled the Center, marking a solid step forward in healthcare cooperation within the SCO. Guided by the principles of multi‐level integration, network‐based collaboration, and intelligent development, the Center is committed to establishing four major platforms. First, an international medical service platform to provide high‐quality and efficient diagnosis and treatment services for metabolic diseases; second, a dual‐track precision talent training platform to cultivate professional teams for disease prevention, control, and management; third, a strategic disease research platform to advance high‐level scientific research and innovation; and fourth, a pharmaceutical and medical technology cooperation platform to promote technological innovation and the sharing of achievements.

To achieve these goals, I would like to propose the following six initiatives:

First, medical training centers should be established across member states to develop a regional talent cultivation system for metabolic diseases and related disorders. These medical training centers will operate under the guidance of the Academic Committee of the China‐SCO Cooperation Center for Metabolic Diseases. Drawing upon the successful experience of the National Metabolic Management Center (MMC) model, the centers will design and develop structured training curricula. At the same time, the programs will be locally adapted to meet the needs of the healthcare systems of different countries, thereby creating an innovative training framework to cultivate specialized professionals in metabolic disease management for all member states.

Second, the establishment of MMCs should be promoted across member states to help improve the regional capacity for the diagnosis and treatment of metabolic diseases. Over the past decade, the MMC “Orange Clinic” model has expanded to 2068 hospitals across China and currently manages nearly 3.5 million patients. MMC has developed a mature system comprising 492 standardized operating procedures and 72 core diagnostic and therapeutic technologies, supported by the MMC 2.0 intelligent management platform with robust capabilities for full‐process data collection, integration, and analysis. In addition, the “Metabolic Integrated Device” developed by our team is capable of completing screening for seven major diabetic complications within 15 min, greatly improving clinical efficiency. Through the MMC Health Cloud System, in‐hospital and out‐of‐hospital management are seamlessly integrated, enabling precise, full‐cycle follow‐up and management of metabolic diseases. Clinical practice has demonstrated that the MMC model can significantly improve target achievement rates in chronic disease management. The glycosylated hemoglobin control rate among patients increased from 22.7% at baseline to 53.2%, while the metabolic syndrome control rate improved from 7.3% to 19.8%, reaching internationally advanced standards. We are willing to share this successful experience with all member states and support each country in establishing localized comprehensive metabolic disease management centers that are deeply adapted to local healthcare resources and population characteristics.

Third, scientific research should be jointly conducted with all member states. Scientific research is the fundamental driving force for advancing the prevention and control of metabolic diseases. We can integrate metabolic disease research resources across SCO member states and establish a multidimensional research framework encompassing epidemiological investigations, comparative clinical studies, and metabolomics research. This framework will support member states in conducting multicenter epidemiological meta‐analyses to systematically integrate prevalence data on diabetes, obesity, and related disorders; organizing multicenter cross‐sectional surveys to clarify the current status of diagnostic criteria, treatment strategies, drug accessibility, and complication screening rates; and carrying out metabolomics studies across member states to identify early biomarkers and potential therapeutic intervention targets. The China‐SCO Cooperation Center for Metabolic Diseases will provide comprehensive support to member states, including specimen testing and personnel training, with the goal of jointly addressing key scientific challenges in the prevention and control of metabolic diseases.

Fourth, a normalized annual academic conference mechanism should be established. The conference will be hosted on a rotating basis by member states of the SCO each year, providing experts and scholars from all countries with a platform for exchanging experiences, sharing achievements, and exploring collaborative opportunities. Through these annual meetings, we will promote the development of a consensus on metabolic disease prevention and control with distinctive SCO characteristics and continuously enhance the overall regional capacity for metabolic disease prevention and management.

Fifth, deeper collaboration at the global level should be advanced. We sincerely invite all member states to join the Diabetes and Obesity Management Association (DOMA) and to establish in‐depth cooperative mechanisms with internationally authoritative organizations such as the International Diabetes Federation (IDF). Within the framework of the World Health Organization (WHO), we will systematically introduce internationally advanced diagnostic and treatment standards, digital management tools, and professional training systems, thereby supporting member states in actively participating in the development of the global diabetes governance system and the formulation of its core agenda.

Sixth, a shared development strategy for the China‐SCO Cooperation Center for Metabolic Diseases should be formulated. By fully incorporating the experiences of all member states while taking into account differences in development levels and cultural practices, we aim to establish an internal regional governance framework and standardized system for metabolic disease prevention and control that are “mutually recognized, operationally feasible, and practically implementable.” This will lay a solid foundation for building a “fair, equitable, universally beneficial, and inclusive” SCO community for metabolic health.

The prevention and control of metabolic diseases remains a long and challenging endeavor. Only through sustained cooperation and persistent efforts can we achieve steady and long‐term progress. The China‐SCO Cooperation Center for Metabolic Diseases serves as a pragmatic platform characterized by openness, inclusiveness, and mutually beneficial cooperation. We are willing to work together with all member states, guided by the “Shanghai Spirit,” to share experiences, promote collaborative innovation, accelerate the establishment of an efficient regional governance system for metabolic disease prevention and control, reduce the burden of metabolic diseases, and improve public health outcomes. Together, we will build a SCO community for metabolic health and make greater contributions to the construction of a global community of health for all.

## Funding

The author has nothing to report.

## Conflicts of Interest

The author declares no conflicts of interest.

